# The Hospital. Nursing Section

**Published:** 1905-10-07

**Authors:** 


### The Hospital.

### Nursing Section.

Contributions for this Section of "The Hospital" should be addressed to the Editor, "The Hospital" Nursing Section, 28 & 29 Southampton Street, Strand, London, W.C.

No. 993.—Vol. XXXIX. *SATURDAY, OCTOBER 7, 1905.*

### Notes on News from the Nursing World.

### LADY HELEN MUNRO FERGUSON SOLVES A PROBLEM.

As the wife of a member of the House of Commons, Lady Helen Munro Ferguson knows that there is all the difference in the world between a select committee reporting in favour of the principle of the State Registration of Nurses and the addition of legislation to the Statute Book. Accordingly, in her contribution to the October number of the *National Review*, which she calls "The Problem of the Trained Nurse," Lady Helen mournfully reminds the advocates of registration that to pass a Bill through Parliament "has now become as difficult a process as the passage of the needle to the proverbial camel." We cannot say that Lady Helen's article is likely to do much towards expediting the process which she fears may be so baffling. At the outset of her summary of events she shows her bias by the statement that it appeared to ardent registrationists "as if the balance of ready-made opinion in the committee leaned decidedly to the side of their opponents." For the rest, from her point of view, she deals with facts fairly enough, and those who share her sentiments, but do not possess either her enthusiasm or her industry, may be glad to be armed with her account of the proceedings before the Select Committee, and the comments which she intersperses in her historical survey. So far as "The Problem of the Trained Nurse" is concerned, she has, of course, only one solution to offer, but in the limited space which she devotes to her own arguments there is nothing either novel or striking—nothing, in short, to influence the "great majority of people" whom Lady Helen Munro Ferguson is afraid will "remain indifferent" to State Registration as a panacea for inefficient nurses.

### FARCICAL NURSING AT CARNARVON.

We direct the attention of the Local Government Board to the serious position at Carnarvon Workhouse as revealed in a letter addressed by the medical officer to the guardians. Having mentioned that an inmate of the hospital of unsound mind escaped from the institution last month, the medical officer affirms that the workhouse, as at present, is not a fit or proper place for the treatment of the unsound of mind. "When there is only one nurse to do the work by day and night," he continues, "it is impossible to treat such cases," and he adds: "The general discipline of the hospital is absolutely impossible, and the nursing of the sick poor a farce." This outspoken communication has been referred by the Carnarvon Guardians to the Hospital Committee, but it appears to us that when the nursing of the sick poor has, in the eyes of their own official, been reduced by those who are responsible for it to the condition of a farce, drastic methods are necessary. The Local Government Board should, we think, at once insist upon the application of the immediate remedy which the medical officer advocates.

### THE DUTIES OF NURSE STEWARDESS.

Commenting recently upon the reticence of the authorities at the headquarters of the Royal Mail Steam Packet Co. with respect to the conditions of service offered to the persons who enter their employ as nurse-stewardess, we advanced in explanation the assurance that the company is inundated with applications for appointments. We ventured, however, in spite of this reticence, to affirm that the woman who undertakes the position of nurse-stewardess has, generally speaking, to sink the nurse in the stewardess. This view is entirely confirmed by the printed statement of the duties which is issued from Southampton to nurses applying for employment. Like the stewardess, the nurse-stewardess has "to attend ladies at 6.30 A.M. and arrange as to what time they will require a bath." She has to take her meals at the head of the children's mess. "After breakfast she should go to the ladies' rooms, make their beds, and generally tidy up." At 11 A.M., "and if required during the day, she will take beef-tea, etc., to ladies, and be on hand about 10 P.M. to get anything required by lady passengers." She has "no scrubbing or menial work," but "is expected to get what she wants from the pantry herself and also return to that place any gear which she may have used for ladies." While she is not to act as children's nurse or lady's maid "to any particular passenger" she is supposed "to render assistance to all ladies who may require her services." The "wages," it is added, are £3 a month, but we understand that no salary is given when the ship is in port. The nurse-stewardess has to provide her own uniform and laundry. From all this it will be seen that the dividing line between the stewardess and the trained nurse who is called by the name of nurse-stewardess is so thin as to be practically non-existent.

### PROPOSED BUREAU FOR THE EXCHANGE OF NURSES.

At an adjourned conference of members of the West Riding Sanitary Committee, and representatives of various other bodies, last week, the recommendations of the committee appointed to consider in detail a scheme for the establishment of a bureau for the exchange of nurses, as occasion might arise, from one hospital to another in the West Riding, were considered, and practically adopted. The County Council have thus imposed upon them the obligation of deciding whether they will undertake the keeping of a register of nurses available for temporary service in hospitals for infectious diseases within the authority's area, the hospital authorities to provide the County Council with the name and qualification of any nurse who can be spared for temporary service in another hospital, and to conduct the negotiations for the loan of nurses themselves. So far as the nurses in the West Riding are concerned, the assent of the County Council will, we believe, be hailed with satisfaction.

### TURNING WINTER EVENINGS TO GOOD ACCOUNT.

The superintendent of an important nursing organisation in the north of England sends us this week an interesting account of the manner in which the nurses working under her occupied some of their evenings last winter. By the aid of the medical man attached to the home a series of illustrated lectures on maternity work were given. There were difficulties at the outset, but these were surmounted by energy and ingenuity, and the animated discussions which followed the delivery of the lectures showed how thoroughly they were enjoyed. In fact, as the superintendent says, every one was quite sorry when they came to an end, and we are sure that the second set which she proposes to arrange in the coming winter will be appreciated. We commend this admirable mode of turning winter evenings to profitable and pleasurable account to the heads of similar institutions.

### ONE MALE NURSE FOR A HUNDRED PATIENTS.

The attention of the Cardiff Guardians was recently called by the Local Government Board inspector to an extraordinary state of affairs in one of the male blocks of the workhouse infirmary. In this block, the inspector stated, there were over a hundred sick and only one male nurse to look after them. The chairman, in referring to the matter, said that the Guardians were "already taking steps to increase the number of nurses." We are glad to learn that since then a meeting of the Workhouse Committee has been held, at which a report from the medical officer on the nursing staff having been read, showing the urgent need of an increase, it was decided to recommend the Guardians to appoint five additional probationers. We hope that the Guardians will make haste to act upon the recommendation.

### MISDIRECTED INDIGNATION.

We print to-day a letter from a trained nurse who is indignant because she saw some nursemaids at an East Coast watering-place masquerading in nurses' uniforms. But her indignation is misdirected. She thinks it abominable that "these little nurse girls" should have the chance of abusing the hardly-earned uniform of a nurse, and writes altogether as if they were the delinquents. We do not for a moment think that they are. The people to blame are, doubtless, the mistresses of the girls who make their maids don the uniform of a nurse because they fancy that it shows the world the superior class of woman they employ to look after their children. The vials of our correspondent's wrath should be emptied upon the heads of these individuals who are largely responsible not only for the abuse of nursing uniform, but are often the cause of its degradation.

### A WORKHOUSE MATRON'S JUST COMPLAINT.

An unusual complaint has been made by the matron of the Stoke-on-Trent Workhouse. At the last meeting of the Guardians she stated that a clerical member of the Board had visited the home at a late hour and while he was there had made inquiries from the subordinate officers as to the times during which she had been absent from the institution. This she described as subversive of good discipline, and we think that harsher terms might fitly have been employed. If there is any reason on the part of a Guardian to suppose that an official is guilty of inattention he is perfectly justified in bringing the question before the Board. He is not, however, justified in assuming the character of Paul Pry and pursuing investigations in the absence of the person he suspects among subordinates whose information may or may not be reliable. In this instance we note with satisfaction that as the result of the complaint the Stoke Guardians passed a resolution expressing their entire confidence in the matron, a form of rebuke which should suffice to put an effective stop to individual espionage.

### THE MISCHIEF AT HULL.

A second effort to obtain for the nurses of the Hull Poor-law Infirmary direct entrance to the Nurses' Home, instead of being compelled to enter it by the workhouse, has, we regret to state, ended in failure. In their very natural indignation the nurses addressed a letter to the Guardians which, if not altogether judicious, contained passages directly bearing upon the issue. They averred, among other things, that women who could not be trusted to behave themselves, were not fit people to be entrusted with the lives of over a hundred sick people; that their off-duty time is so limited that even the extra five minutes occupied by going round by the front is a consideration; and that they object to pauper inmates being trusted to book them in and out at the lodge. We think that they should not in this letter have alluded to any of the Guardians by name; but on the main question our sympathy is, of course, entirely with the nurses. In proposing a resolution intended to set aside the decision on which we commented a fortnight ago, Mr. Councillor Pybus pointed out that if the Guardians insisted upon putting a wall round a part of the workhouse with the intention of stopping the nurses from going out, they would expose themselves to strictures from the general public. This may be true, but the main point is that the determination of the Guardians, by a majority of four, to persist in their action, will inevitably tend to promote discord in the institution under their control.

### MORE NURSES FOR THE MIDDLE CLASSES.

Another attempt is to be made in London to organise a staff of visiting nurses for the middle classes. Mrs. Lewis-Hill—better known to the world as Mrs. Ada Lewis for her admirable philanthropic work in Paris—who proposes to open an institute for the supply of this class of nurse at the end of the present month in Oxford Terrace. The nurses in this case are said to be intended for "the poor of the middle classes." As no appeal is made to the charitable on behalf of the new movement the promoters, of course, have a perfect right to fix what scale of fees they please. The minimum fee is to be 5*s.* per week, and the maximum fee 10*s.* Whether on this basis it can be made self-supporting remains to be seen. We understand that a lady superintendent and two nurses have been engaged.

### AN UNUSUAL TESTIMONIAL.

There was an animated discussion at the last meeting of the Newton Abbot Guardians concerning a testimonial given by the medical officer to a nurse who recently left their employ. It seems that certain complaints were made against the nurse and she resigned. When the committee had the resignation before them, but for the advice of the medical officer, they would have recommended that she should be granted a testimonial. As it was, they made no recommendation and the Guardians granted the nurse a certificate for the period she had been in their service. Subsequently, it came to the knowledge of the committee that the doctor had given her a testimonial and the chairman of the committee warmly protested at the meeting against this action after he had impressed on the committee that the nurse was not fit for her post. The medical officer, called upon to explain, admitted that he had given what he described as "the sort of thing you would give to a domestic servant," not testifying to her capabilities or that she was a good nurse. A motion that the explanation be accepted was met by an amendment regretting that under the circumstances the medical officer should have given a testimonial or certificate at all. Eventually, however, the order of the day was passed by 24 votes to 17. The testimonial given by the medical officer may have been correctly described as "colourless," but we think that the course he pursued, though condoned by the Guardians, was, at any rate, unusual.

### BIRMINGHAM NURSES AND THE PENSION FUND.

On Thursday last Mr. Louis H. M. Dick,, Secretary of the Royal National Pension Fund for Nurses, delivered an address in the Lecture Theatre of the General Hospital, Birmingham. The audience numbered over seventy, and although the attendance, through various causes, did not reach the total anticipated, it was thoroughly representative of all the hospitals and nursing institutions in Birmingham. Among those present were Miss M. E. Jones, Matron of the General Hospital; Miss Elkington, Matron of the Queen's Hospital; Miss de Chastelain, Matron of the Jaffray Hospital; Miss Bell, Matron of the Children's Hospital; Miss Ewing, Lady Superintendent of the Birmingham and Midland Counties' Institution for Trained Nurses; and Miss Hayward, Superintendent of the District Nursing Society. Mr. Howard J. Collins, House-Governor of the General Hospital, having introduced the Secretary of the Pension Fund to the audience, [illegible] Dick reminded his hearers that nearly two years had elapsed since he last had the pleasure of addressing the nurses in Birmingham from the same platform. In the interval the Fund had made very great strides, having issued just over 2,000 policies. Its invested capital had increased by over £170,000, and now stood at the very satisfactory total of over £970,000. Mr. Dick explained why it was imperative that nurses above all others should exercise thrift, and how essential it was that they should choose an absolutely safe method of providing for their future and he urged that the Pension Fund was peculiarly adapted for the purpose, possessing advantages not approached by the commercial insurance companies. On behalf of the Council, Mr. Dick thanked the authorities of the General Hospital for giving him the use of the Lecture Theatre. After the address, by the courtesy of the Matron and House Governor, tea was served in the Board-room.

### THE NURSES' QUARTERS AT THE NEW LEICESTER INFIRMARY.

The provision made for the nurses of the North Evington Poor-law Infirmary at Leicester, which was opened on Thursday last week, are of a very complete and up-to-date character. As in the wards of the infirmary, the whole of the furniture is of oak. Each nurse has a separate bedroom fitted with a combination washstand and dressing-table, a wardrobe with long mirror, linen basket, and chair. In the recreation-room there are numerous easy chairs and chesterfields, and a piano. The nurses have also a library and study fitted with book-cases. The latest text-books are supplied for their use, and in this room they have a skeleton, a disarticulated skeleton, a female pelvis, and a fœtal skull. The course of training is three years with a further period of six months for midwifery if required. Lectures will be delivered by the medical men weekly, and massage, sick cooking, and dispensing will be taught. A very liberal dietary scale, with an additional allowance of fresh fruit and vegetables has been granted by the Guardians, and such discretion has been vested in the Matron that there can be no danger of monotony in meals. The laundry block is connected by a covered way with the matron's department and is so arranged as to facilitate easy supervision by the head laundress and the proper circulation of the clothes from reception to delivery.

### RATIONS IN IRELAND.

The Ennismore Guardians recently employed a trained nurse to attend a fever patient at night, her remuneration being 11s. 6d. a week and food. It cannot be said that the allowance of food was wanting in liberality. At a meeting of the Guardians it transpired that the rations of the nurse for a week consisted of seven pounds of bread, seven quarts of milk, half a pound of tea, a quarter of a pound of coffee, two pounds of sugar, three pounds of fresh meat, three pounds of bacon, one pound of sausages, one pound of tripe, two pounds of marmalade, three pounds of biscuits, one pound of rice, one pound of tapioca, one pound of cornflour, one pound of cheese, one chicken, one fish, two dozen of soda water, one cake, two pounds of fruit, and one dozen and a half of eggs.

### The Nursing Outlook.

"From magnanimity, all fear above;
From nobler recompense, above applause,
Which owes to man's short outlook all its charm."

### THE DISTRICT NURSE IN SCHOOL AND HOME.

We recently pointed out how much district nursing means to the poor, the doctors and the nurses themselves. There is however a further and perhaps a more important class, the children, on whom the district nurse may confer inestimable benefits. It is only a few years ago—to the credit of women members of Boards of Education be it recorded—that the trained nurse was introduced into the public schools. The movement originally commenced, we believe, in New York City, and it is interesting to note that after a few years' experience, the New York Board of Health was able to report that some ninety-eight per cent. of cases of exclusion of children from the schools was due to diseases principally of the eye, or external body, of such a nature that, when proper attention was persisted in until a cure was effected, there was no need for barring the child from school attendance. In consequence of this experience on the part of the medical officer, and the introduction of the district nurse into the schools, Dr. Newmeyer states, as a result of his experience, that the nurse in a school population of 157,500 children would reduce the exclusions from 7,600 to 600, seeing that 7,000 of them suffering from various ailments could then remain at school, or lose only a day or two away from school and education. Dr. Newmeyer states his views in a very able and convincing report, and the result of their being put into practice in Philadelphia was a reduction of the numbers of children excluded and of truant cases to a total which is wholly insignificant compared with the numbers formerly absent.

The district nurse attached to a school has handed over to her the case of every child excluded for disease. These exclusions are necessary to obtain the co-operation of parents. It is the nurse's duty to see that no children are medically treated by the school physician where the family have a doctor of their own, and the nurse arranges that the parents lose no time in consulting their medical attendants in these cases. In practice it is found that children excluded for uncleanliness in great cities may wait a week before they receive the necessary bath. Where a nurse is employed, however, she pays a visit to the home, which results in the child being properly washed and attended to without delay.

We have heard a great deal about the ignorance of poor mothers in regard to the feeding of children and infants, but their ignorance in regard to cleanliness is quite as great if not greater. Many mothers do not know how to bath their children. A nurse found one mother giving what she called a bath to her baby. The mother had a little cold water in a tin basin, but no soap, and she merely dipped her hand into the water and then rubbed it on the baby. After she had rubbed it all over in this way she took a very dirty cloth to dry it. In this family all the children were washed in like manner, if at all. Even in cases where the parents have more intelligence, where no nurse is employed dirty children become subjects for re-exclusion in a very short period. The advent of the nurse, however, by teaching the parents how to attend to their children, results in a permanent improvement, and so renders re-exclusion practically unnecessary. In cases where children are excluded for a contagious skin disease, the services of a nurse are essential. Without her aid several days are allowed to elapse before such a child enters a dispensary for treatment. Parents in such cases often wait for nature to cure the child, especially where they cannot afford to pay the medical practitioner. This is the case because the parents having other small children cannot spare the time to sit for hours in a dispensary to wait their turn. The evils attending this practical difficulty are well illustrated by the case of a child who had several corneal ulcers which threatened to destroy the child's sight. In this case, notwithstanding its seriousness, the parents gave no attention to the child, even after it was excluded from school attendance. When the nurse saw and remonstrated with the parents they said they had no time to have the child attended to. In this case the nurse herself took the child to a dispensary, where a cure was speedily effected and so the child's eyesight was saved. We have thought it well to give practical illustrations of this description, in order to direct the attention of the education authorities in London to the importance of providing for the attendance of a district nurse at every public school.

It may be remembered that in July 1904 the Education Committee of the London County Council was authorised to appoint a staff of twelve nurses working under its medical officer, to visit the elementary schools. The result of the work done by these nurses has been most satisfactory, but they are too few in number to adequately accomplish the work which ought to be undertaken in the interests of the education, as well as of the health of the children. Experience shows that it is found that parents do not attend to their children's ailments, with the result that the period of their absence from school is indefinitely prolonged. The school nurse supplies the remedy. She follows the excluded child to its home and sees that its ailments are properly attended to. She obtains the co-operation of the parents in maintaining the cleanliness of the child, and securing its general health, with results which are permanent. The doctors are thus able to leave at school many minor cases that require attention, instead of making children lose their school time, and teachers can be sure that pupils that are excluded by the doctors will be followed up, cured promptly, and so miss the least possible time from their classes. In this way the expenditure of a relatively moderate sum upon the employment of an adequate number of nurses in the public schools permits the thorough education of the poorer children which would otherwise be unattainable.

### Medical Electricity and Light Treatment.

By Kate Neale, Sister-in-Charge of the Actino-Therapeutic Department, Guy's Hospital.

X.—FINSEN LIGHT (*concluded from p.* 415).

IV.—**After-Treatment.**—The effect on the skin of an hour's application of the Finsen light is much the same as exposure to a bright sun—namely a blister. The blister appears from six to twelve hours after the treatment, and must be protected from injury and dirt by a dressing. The most suitable consists of ointment (the doctor will prescribe it, usually boracic or zinc) spread on lint. A small circle of this should be cut out just large enough to cover the spot, but before putting it on wash off the blue pencil mark with sesame oil. Having finished with the patient, thoroughly carbolise the pressure-lens, and polish it with methylated spirit and a soft cloth. Rinse your own hands in some antiseptic such as lysol, and wash them well with soap and water. The dressing must be changed three times in the first four-and-twenty hours, and thereafter twice daily. Impress very emphatically on the patient the importance of keeping the part scrupulously clean, and direct him to bathe it with warm boracic lotion, or hot water each time it is redressed. Only by such care can the full benefit of the treatment be assured and the appearance of fresh scabs avoided.

When the patient next comes up for the treatment the blister should, if necessary, be cut or pricked to allow its contents to escape, placing wool to absorb the fluid. For a few days there is a watery discharge from the raw surface, but in a week or so the area scars over and heals. Occasionally this "reaction" as it is called is less vigorous; no blister forms, and some redness and swelling alone mark the application of the treatment. Such a result must be considered unsatisfactory, for the production of a definite blister should be your aim. It is generally best not to reapply treatment to the same area until the reaction has quite subsided.

V.—**Special Practical Points.**—(a). **Treatment of Eyes, Nose, Chin, etc.**—If the lupus patch is in the neighbourhood of the eye, protect the patient's sight from the lamp's glare by dark spectacles, or better still by covering both eyes with a double fold of brown paper, or the ordinary red paper used by photographers.

When the surface of the nose is to be treated, plug the nostril on that side with wool wrung out of boracic lotion. In this way a firm resistance is obtained to the pressure of your lens. Similarly when treating the lower part of the face fill in the interval between lips or cheek and teeth according to the site of the disease.

Areas under the chin are awkward to get at and the couch has to be placed immediately beneath the light. Under such circumstances there is a risk of the patient's clothing being burnt by white-hot particles which occasionally splutter off the carbon points. There is usually, as shown in the frontispiece, a metal tray fixed immediately beneath the light to catch these sparks, but, for proper safety, the patient must be covered with an expansive leather apron.

(b). **Removal of Dressings.**—If the dressing ha got dry and adheres to the skin, moisten it wel with boracic lotion before attempting its removal.

When changing a dressing lift it off from both sides simultaneously. If you peel it off from one side and there is a tag of loose skin beneath, the result of a blister, you will cause unnecessary pain.

Remember that as soon as the dressing is removed and the blistered surface exposed to the air, pain and burning quickly begin. Therefore, when a patient has many dressings, change only a few at a time (say half-a-dozen), and re-cover these again before exposing more.

The neatest way of retaining a number of circles of lint on the face is to cover them with the ordinary surgical chin bandage. Take a piece of wide bandage, about a yard in length, and split its two ends for ten to twelve inches. Place the middle of this bandage either on the point of the chin or beneath the jaw, and tie two ends together on the top of the head and two behind the ears. Finally, tie the ends of the two knots together.

(c) **Other Practical Points Worth Remembering.**—If the spot to be treated is smaller than the focussed light of the lamp, protect the area around with red photographic paper.

A difficult but not uncommon site of treatment is the angle between the eye and nose. A special pressure-lens is made for this area, but generally speaking any irregularity of surface can be levelled by packing behind the pressure-lens, between it and the skin, a piece of wool wrung out of boracic lotion.

A patient will sometimes complain during treatment that the rays produce a burning feeling. This generally means that you are using your pressure-lens too gently. A firmer and more general pressure will make him more comfortable. But if the pain still persists replace the cap on the telescope and proceed to add more packing beneath the lens.

### The Finsen-Reyn Lamp.

Practically everything I have said in the last few pages applies equally well to treatment by the Finsen-Reyn Lamp. There is the great advantage, however, that this lamp is movable, and this facilitates the arranging of the patient as well as the focussing of the light. The carbon points are kept in position by an automatic regulation, but it is nevertheless very necessary to see that they do not lie too near the lens else the latter will crack. The pressure-lens is used exactly as with the Finsen lamp, but the light is not focussed to quite so small an area and the floral design should cover approximately the size of a shilling.

### French Lamp (Lortet-Genoud).

With this lamp the patient usually sits in a chair and presses his face or whatever part is to be treated against the rock-crystal lens in the shield. No pressure-lens is used, but the customary precautions must be taken to start the circulation of water before the light is switched on. The carbon points are brought close together by turning the handle (A) so that they grow white-hot and a brilliant glow appears between them. If they begin to flare at all they must be brought nearer. After each treatment the carbons must be "repointed" (*i.e.* readjusted).

**Ultra-Violet Lamp.**

The description of this lamp given on page 400 will sufficiently explain its use. The lens in the handle acts as a pressure-lens, and no focussing has to be done. The very superficial action of the lamp limits its usefulness.

### The Nurses' Clinic.

### THE DISPENSARY. BY A CERTIFICATED DISPENSER.

### HOW TO FIT UP AND ARRANGE A DISPENSARY.

A dispensing-room should not be too large, as a good deal of time can be wasted by wandering from one end of the room to the other to fetch bottles or appliances. It should be compact and fitted with plenty of shelves and cupboards: a poison cupboard and a lead-lined sink, with running water, hot and cold, and fitted with a waste-pipe. If possible, it is best to have one tap of water connected with the main and another tap from some other source, so that if, for some reason or other, communication with the main is interrupted the other tap is ready to hand. Nothing is more annoying to a dispenser than to find the water-supply cut off for an hour or two owing to repairs which necessitate turning off at the main. A Bunsen burner is also required, and a gas-fire in the grate is better than an ordinary fire, as it can be quickly lighted and saves much trouble; a small pair of light steps for reaching the higher shelves, a kettle, a saucepan, cork-presser, ointment slab, funnels (enamelled ones are best, as they are unbreakable and not affected by corrosive liquids), infusion jugs, steel and bone spatulas, a large pestle and mortar for ointments, a medium-sized one, and a little one, one or two pint jugs, measure-glasses for measuring ounces and drachms, and a couple of minim glasses, one large set of scales and weights for weighing by the pound, and a small set for ounces and grains, stirring rods, scissors, filter paper, bottles, and tow complete the list of necessities, though many more things might be added according to the purse and inclination of whoever has the fitting up of the dispensary. A pill machine is unnecessary, as pills are usually bought wholesale and not made in a small dispensary. It is best to have stock bottles of a good size, as much time may be wasted by having to constantly fill up small ones. Syrups should be kept in dark-blue glass bottles fitted with a plug; poisons must be kept all together in a cupboard specially assigned to them, and substances that spoil or turn colour by the action of the light, as santonin, silver nitrate, and prussic acid should be locked up in the dark. In some dispensaries a few of the poisons, as Liq. Arsenicalis and Liq. Strychninae, are kept on the shelves mixed up with the other bottles, but it is far better and safer to keep all the poisons safely locked up together in one place, and it also lessens the risk of anybody getting hold of a poison during the dispenser's absence. Two or three large earthenware jars with taps are very useful for keeping infusions when large quantities are used, and a large glass jar, also fitted with a tap, for cod-liver oil will be found very convenient.

The bottles containing liquors, tinctures, etc., should all be of the same size and labelled alike, otherwise a patchy and untidy appearance is given to the room. Labels written by hand on paper soon get dirty and untidy; indestructible ones can be made with little trouble with spoilt photographic films cleaned and fastened to the bottle with diamond cement; the label is stuck to the bottle with the film over it. The bottles containing powders should be of a uniform size and labelled in the same way. Pint bottles are good for keeping stock solutions in, and all the bottles should have glass stoppers instead of corks. A very good appliance for removing a stopper which has become fixed in the bottle, is made of wood with different sized holes cut in it to fit various stoppers, which can then be generally got out with little trouble. Another good plan is to gently tap the stopper with a piece of metal, a steel spatula is generally the most handy; or a cloth wrung out in boiling water and wrapped round the stopper and neck of the bottle for a few minutes is often found effective.

The shelves should be made a little wider than the bottles, ensuring only having a single row, as when there are two rows on each shelf it takes up more time in getting at the back ones (and in a busy dispensary every minute is of importance) or else the bottles which are placed at the back are apt to be overlooked or forgotten. It is not a good plan to have many shelves high up and out of reach, as it adds much to the fatigue of the dispenser if the steps have to be constantly used. The shelves are best made of plain wood, unpolished, and they should all be well scrubbed at least once a week. The floor should be either of plain boards or covered with linoleum or oilcloth, which may be washed over every day in addition to the weekly scrub. The sink must also be thoroughly cleansed daily, and hot soda and water poured down the drain helps to keep it clean and nice, while the entire sink should have a good cleaning with chloride of lime every now and then; if this is done over-night the unpleasant odour of the lime will have disappeared in the morning. The dispensary counter should be about the height of an ordinary table, and should, like the shelves, be of plain wood, or it can be covered with white American cloth; it gives a better appearance to the room to have the shelves and counter so covered, and it is preferable to putting paper on the shelves which soon gets dirty and crumpled. A nice large piece of marble let into one end of the counter is really an improvement on separate ointment slabs.

The space under the dispensing counter should be occupied by cupboards and drawers; one drawer to be used for keeping plasters, filter paper, the prescription book, spatulas, etc., in, while a separate drawer for different-sized willow ointment boxes and pill boxes is essential, as if all the boxes are kept in one drawer a good deal of time may be lost in hunting for the required size. It is much more convenient to keep certain powders—as mag. carb., sodii bicarb., or ammon. carb., or anything else used in large quantities—in drawers rather than in the large bottles in which they come from the wholesale druggist. Two or three more drawers will be needed for various-sized corks, while another one should be kept apart for the dispenser's private use. After filling up the bottles on the shelves, the bottles containing what remains over of the bought drugs are kept in the cupboards, and the roots and chips used for making infusions and decoctions can be placed there if there are not sufficient drawers to accommodate them. If there is space, it is very handy to have a big cupboard with several shelves in which to store reserve drugs, pestles and mortars, and other appliances, and a supply of measure glasses besides those in actual use, which should be kept ranged on shelves over the sink. A few shelves under the window, or under the sliding door through which the patients receive their medicine are useful on which to keep graduated bottles. For the labels, strips of leather may be nailed on either side of the sliding door, with

**THE NURSES' CLINIC**—*Continued.*

brass-headed nails at intervals to make divisions to slip the labels into, or a better plan is to have narrow bits of wood, little more than the width of the label, fastened along the door, and divided into compartments and painted the same colour as the other wood work. The placing and arrangement of the bottles is best done on a systematic plan, and one that a stranger can easily grasp on coming fresh to the work; perhaps the best way is to keep all of a kind together, as all the syrups, all the liquors, tinctures, etc., and have them each in alphabetical order, always keeping the stock mixtures and other much used bottles nearest to hand on the lowest shelves and the less used ones higher up.

### Incidents in a Nurse's Life.

**Contributions for this column are invited.**

### MY FIRST NIGHT ON DUTY. BY THE MATRON OF A FEVER HOSPITAL.

It was 9 o'clock p.m., the last Saturday night in August, and I was, for the first time, on night duty. The senior day nurse had just left me standing in the corridor outside the door of one of the four wards of the male medical landing. I had had five minutes of instructions and a grim reminder from her that there were *five* of my cases which might die "that night." No. 64 had been awfully bad all day; 71 had been pronounced dead just as the nurse had been busy with the teas in the afternoon, but had come round again; and the others wanted special attention too. My poor brain worried itself with all the future that one night was capable of holding for me. I gave one longing gaze at the white of the nurse's apron as she passed along the corridor, and then, with a final warning "to watch 71 well between three and four o'clock," she disappeared down the staircase out of my sight. I could not help thinking, "How shall I feel when I see her next?" I turned into the ward where 64 and 71 tossed and moaned in a weary battle, with all the odds against them, against the grim warrior Death. I looked at 64's chart; his name was Gilmour, printer, suffering from heart-disease and its usual complications. "Gilmour," I said quietly, "what do you want, my poor fellow?" He had his feet out of bed, and as I spoke I gathered them in under the clothes. "What do I want?" he gasped. "I want life, that's what I want," and he lay back wearily against his bed-rest. I glanced at 68, a case of dropsy; he, too, was getting out of bed. "Don't get out of bed, the floor is so cold for your poor swollen feet. Have this nice drink," and I persuaded him to finish the milk from his cup and to settle back again. 71 seemed quiet, and I felt glad, for he was a terrible case of aneurism.

Now I must see the men in the other wards. I glanced at my watch; it surely must have stopped, for by its showing I had been less than a quarter of an hour on duty. Oh! what an eternity the night seemed to look forward to! My watch was right, for somewhere one of the city clocks chimed the quarter, and I swallowed a sickening sigh as I glanced all round the ward and passed out of it into the other. One young fellow was dying of phthisis; he said something, and I went over to his side.

"I should like to see my mother, nurse."

"All right, John," I said. "I'll get the sister to write for you in the morning. Try and go to sleep now."

I knew his mother would not be written to, for she had died since her son came in here, from an accident. I had heard the nurses talking about this lad at tea-time, as nurses will, and I knew how impossible it was to tell the dying boy such news. "He will see her some time soon," I thought, "and it will be all right."

I gave John his dose of medicine, saw the other men were seemingly comfortable, and went round the remaining two wards. One old man was pulling at his locker, and I hurried over. "What do you want?" I scrutinised his name on his chart: Terence Kavanagh. "You are making a dreadful noise, Kavanagh. Can I get anything for you?"

"You can that, miss," he said in a low voice. "There's tuppence rowlin' about somewhere in my ould clothes, and I thought maybe you'd run out unknownst for a bottle of porther, and we'd have it between us, and no one id be the wiser."

Shades of Temperance protect me! Was ever such an offer made to a shining temperance member before?

"The shops are shut, Kavanagh," I said. "Wait until they open in the morning."

"All right, miss, I'll thry and wait; but you won't forget."

"I'll not forget, Kavanagh."

Then I looked at my direction sheet; no poultices until 10 o'clock. A weary sound of difficult breathing, sharp moans of pain, coughing, snoring; this was to be my portion for that night, and many successive ones, until the two months were up; this was to be the chief music in the air for me. But the superintendent came round as I was making a poultice for an old man in the corner bed, and she very kindly helped me to apply it. I was speechless with amazement, as, judging from the other nurses' chatter, she did nothing but glare at one, and make one terrified into doing a thing wrong through nervousness.

"How is 71, nurse?" she asked. We went to his bedside.

"Seems easier than he has been all day. I hope he will have a good-night," and I was left alone once more with a landing full of moans and groans.

The night sister came round before midnight. The usual question, "How is 71 to-night?" "Seems sleeping, sister," I said, and having gone round all the wards—my wards now—she, too, went off. I was seeing to the fires when I thought I heard a sharp quick call of distress. "That is 71," I concluded, and I hurried to him. He was out of bed in an instant, standing at the foot, grasping the rail, and, oh! the dreadful gasping, the outward sign of his sad sufferings! I flew out and summoned the night sister, and she hurriedly came to him, a minute or two later calling the resident medical student. He administered a nitro-glycerine tablet, but the poor fellow seemed to get but little relief, until the beautiful sun shone in the windows just at 6 a.m. Sunday morning, too, and bells were chiming, and the sufferer looked up from his stand at the bottom of the bed, "Thank God," he said, "for another day." I coaxed him at last to crawl back to bed, and propped him up, and once more a little respite came in the shape of sleep. As soon as I could I went to the wardmaid, cautioning her not to make a noise with her grates, as "71 had had such a fearful night, and he was dozing now."

I felt, like him, indeed, grateful for a new day, for I was very tired, and I was glad to know I could go to bed as soon as we left the dining-room at 10 o'clock. When getting the stores for the day, the superintendent asked me if I was very tired with my first night on duty. But I felt so glad that I had got through the night without making any very serious blunder, and the knowledge that bed-time was near, combined with the consciousness that I had a letter and a parcel from home to take to my room and revel over, did so much to buoy me up that I only replied "a little" as I registered a resolve that I would go on and not give in. As I dropped off to sleep I told myself that all would be well, for it was nice to know that I was not leading a useless life, if I but tried my very best to prove a blessing to the poor wearied sufferers placed under my care.]

### A School in a Hospital.

School in a hospital! Yes, a real school. There are the lesson-books, needlework, note-books, and pencils; there are the teachers with voice and hand engaging the attention and drawing forth the answers of the children. But there are no desks, no inkwells here, for the scholars, poor children, are in bed, not even sitting up to write, but lying flat upon their backs, their legs—with diseased hips—kept in position by a cord and weight attached. Of varying ages they are, from twelve down to three years old, of both sexes. Children who have been to school before the joint became diseased, children who have never learnt. No easy task to keep them all employed on tasks of varying kind. But the teachers, only two among some sixty children, accomplish it. And such attention, such quiet as never yet was known in school or hospital ward! Yet it must not be thought that they do not like their lessons. At first, maybe, after months of doing as they liked, subject only to the nurse's admonitions, talking, singing, laughing at the top of their voices, they found it hard to keep silence. For a little, after constant change of occupation, exchange of books and toys and drawing-slates, they found it hard to settle down to regular work, to submit to discipline, and concentrate their attention on the task in hand. But now, even after a month only, they would miss their work and find the time hang heavy on their hands without the definite occupation which their lessons provide.

As we go from one ward to another, two large, two small, we listen and watch. Those children are saying their "twice times table," and on the balcony, where some eight or nine cots have been placed in order that they may breathe the fresher air, a group of little patients are repeating some verses. Wait awhile in the ward and you will hear them sing "Little Bo-Peep," the teachers helping the unaccustomed voices with the accompaniment of the piano. Those bigger boys have written up their diaries and submitted them to the teacher's scrutiny, and now they are preparing from one of the charming historical readers a lesson from a page of England's history, the battle of Crecy and Poitiers.

In the morning, after a Scripture-lesson, hymn-singing, and prayers in each ward, such tasks as these are in progress, or the little ones are making "pothooks and hangers," the smaller children "summing" or listening to an object lesson, the elders doing dictation or learning from the geography book. In the afternoon there will be a change of occupation; the bigger girls will have embroidery or lace work to do, the elder boys macramé work, or maybe bent-iron twisting, the little ones some kindergarten task with coloured beads or papers.

Then there are physical exercises; but only the arms are raised and dropped in time, the hands twined and the fingers extended together, for these little children cannot use their feet. But breathing exercises, too, are taught, and great good must result from the expansion of the contracted chests, the inhaling of the fresh air that blows in through the large windows from the green, shady square opposite.

It is quite a revelation to a visitor—the control the teachers have, their quickness in changing and arranging the tasks from time to time. Now one will stand in the centre of the ward, teaching the children a song, then quickly passing from bed to bed, correcting an exercise, setting a sum, and taking up the next line of the piece of poetry or nursery rhyme which is being taught to the little ones.

And without causing any interruption, while the lessons are going on, there is the maid on hands and knees blacking the grate. A nurse is polishing the brass knobs upon the beds, and the staff nurse and sister are dressing a wound and bandaging a shrunken limb.

And where is all this to be seen? At the Alexandra Hospital for Children with Hip Disease, in Queen Square, Bloomsbury. And the project started as follows: The chaplain, in his weekly visits, had noticed the aimless lives of the children in the ward, their want of regular occupation, the danger they were in of being spoilt, the noisy shouting and singing, and the reading of nothing but story-books. Here was an opportunity—wanting in the ordinary children's hospital—in the fact that the patients would be in most cases not months, but years under treatment. Could not something be done to restrain their wild spirits, to give them more profitable occupations, to develop their often precocious talents, which might be turned to good account on their leaving the hospital?

He noted how some of the lady visitors had taught the children to make baskets and string frames, helped them to read, or to write and sum; but so little could be done by occasional visits and with no proper system or discipline. And it occurred to him that a school might be started, with fewer hours of study than in the elementary schools, and with methods suited to the ages, capacities, and peculiar circumstances of the scholars. The approval of the doctors was gained for the idea, the hearty co-operation of the nurses was assured. Then followed an interview with H.M. Inspector, Dr. Eichholz, a well-known authority upon school hygiene and the training of defective children, more than one visit from Mrs. Burgwin, the lady inspector of the London County Council to the Invalid Children's Schools, and a scheme was drawn up and a time-table prepared. Some time elapsed before suitable teachers could be secured, but at last they came, school materials were provided, and each day, with the exception of Wednesday afternoon—when the parents visit—and Saturday, the school is in full working order and accomplishing a quiet work, not only for the children now, but as helping to fit them for the time when they must leave the hospital and make their way in the world, hindered by lameness in many cases, and in others by a delicate constitution.

Such is the school in the hospital, and a visit, moreover, to the wards between two and four any afternoon would be sure to enlist the sympathy of the visitors in the children, and arouse their interest in this beautiful hospital, which is doing so great and blessed a work for suffering childhood in an out-of-the-way corner of Bloomsbury.

### Five Months in a Maternity Hospital.

After three months in a general hospital I came to the determination, at the end of that time, that I should not be strong enough for three years' training. So I decided to become a midwife, and applied to several lying-in-hospitals for particulars of training.

Eventually I made a decision, and on one bright day in May I arrived at the hospital to take up my duties. On arrival I was shown into a large bright room (which I afterwards found out was for the nurses' use), and in a few minutes the page came to summon me to the matron's room.

The matron received me very kindly, and hoped I would do my best to do the hospital credit, and after a few more instructions she rang the bell for the maid to show me to the dining-room.

Here I found 30 or more nurses seated at tea, looking cheery and well, and quite at home. As I took my seat I felt that amongst so many bright faces one could not fail to be happy.

During tea I made friends with a nurse who had been in hospital one week, and she told me that she was quite contented, and assured me that I should soon be so too.

I had been told by the matron to go upstairs to the second floor after tea, and there I made my way.

The First Start.

On inquiry I was taken to the sister's room, and after asking me a few questions, and carefully studying my appearance, the sister told me to go to the floor kitchen (there was a small kitchen on each floor) and help the nurse prepare the patients' bread and milk for supper. Later on I was taken into several of the wards, and allowed to help the nurses. Thus I started my first insight of maternity work.

At 8 o'clock a bell rang for supper for the first batch of nurses, and at 8.30 the rest went down. At 9 p.m. we went down to the chapel for prayers, and afterwards we went to our respective cubicles. At 6 o'clock next morning the rising bell rang, and springing out of bed, and opening my door, I inquired of a nurse who was passing what it was for, and was informed that it was the bell for rising.

As soon as I had hastily dressed I followed several nurses into the wards, and was met by the staff nurse, who told me to follow her. I found myself in a large room, with metal and china utensils on long shelves, and here I was told to clean the brass scales and the weights used for weighing the babies. I afterwards found that my duties included the polishing of two metal bed slippers, teapot and kettle, and at 10 o'clock each morning, when all the wards, etc., were in readiness, you would have thought that all the utensils in this particular room were of silver, they shone so brilliantly.

Each nurse has to look after two patients and two infants—unless, of course, she may happen to get twin babies, or the infant dies at birth—in a ward to herself. Or she may nurse in a double ward, with four beds and four infants, one nurse for every two beds.

All the wards possessed polished floors; white enamelled beds, covered with scarlet counterpanes, and prettily draped cots, each in readiness for its tiny visitor; fires burn brightly in each ward, day and night, all the year round, and smart fire-guards with brass rails surround each hearth, whilst on a trivet stands a bright singing kettle always ready for use.

Small polished lockers, to hold books or any presents friends may bring for "baby," are also beside each bed, and last, but not least, two soft, cosy armchairs for the patients when they are able to get up.

Later on in the day I was called to the labour wards to see my first cases through, and I went, feeling very nervous and very awkward. However, the labour sister was very patient, and showed me how to bath one infant, who weighed 13 lb. Oh, how he did roll and cry! and I feel sure that he knew he was in inexperienced hands by his very behaviour. It was with a great sigh of relief that I at last laid him carefully in his cradle, near the fire, and commenced clearing up.

The second patient's baby I managed better. It only weighed 6 lb., and was much more at home with me, and it neither rolled nor cried, so with great pride I found myself the happy possessor of two patients and infants in a dear little ward to myself.

How I got through those two cases I hardly realise now, but I shed more tears and had more scoldings than ever I had had in the whole of my life before, and although at the time I felt I did not deserve it, I can quite sympathise with sister now.

In the Labour Wards.

The next two I had I got on well with, and as I liked my duties I really did improve, and when at the end of three months I was told that I was to be transferred to other work I felt quite sorry. Matron presented me with a badge later on in the day, and I was told to go to the labour wards. Here I was taught to deliver, to manage cases before, during, and after confinement, the care of infants, etc., and we had to make the beds aseptic, to see the bedding stoved, to keep the linen cupboards tidy, and to keep the door-handles, taps, slippers, douche cans, etc., bright. We also learnt how to sterilise. We were quite proud of our work, and loved to keep everything nice, so that we might deserve the encomium of visitors going over the hospital, who would often remark, "How beautifully everything is kept here!" It was wonderful how those few words repaid us for our trouble. After four happy weeks I was put on "district" under a registered midwife, and I had many experiences. The first night I was called out to a case quite one and a-half miles away, and went in company with the midwife. On arrival I had quite made up my mind that we should find nothing ready for our use, but in this case we were extremely fortunate, for there was a fire, clothes, and food. In addition to the patient we discovered three children in another bed. These we handed over to a neighbour to take care of. Then, having got out lotion, etc., we proceeded to examine the patient's clothing and bedding, which was luckily clean, so that we could prepare for the delivery, which took place an hour later. The "little man" weighed 9 lb., and we called him, for fun, "Jumbo.' I afterwards heard that he was christened "Baden Powell."

Another case we visited was quite destitute, and, as the mother had nothing whatever to dress "baby" in, I eventually cut two holes in my flannel apron and wrapped him up in it. Later on I was able to obtain the use of the district maternity bag for the case. An experience not likely to be forgotten was our arriving at a case to find the baby already born, and with a large tumour on its right side. The "lady-in-waiting" was crying, because, she said, "the midwife would think, when she saw the 'lump,' that the baby had been dropped, and really it wasn't her fault," etc. We had to reassure her many times that we knew she was not to blame before she would help us in any way. Even the doctor, when he arrived to see the child, was met with more tears and protestations, but we eventually succeeded before leaving the case in making her smile again.

My examination, which came later, seemed to me a stiff one, but I got through, and I think it brought, maybe, greater happiness and pride when I was presented with my —in my opinion at least—well-earned certificate.

### Among the Hop-pickers in 1905.

BY ONE OF THE NURSES.

It was with a feeling of determination to see the rosy side that I entered upon my first experience of nursing the hop-pickers. The engagement practically settled, I mentioned the fact to my acquaintances. They at once tried to paint the life in its darkest colours; but I was fully determined to face all ills, and treated all their remonstrances as a joke. The time arrived and I entered upon my duties. But I looked in vain for the dark side and found instead one of the happiest experiences of my life.

The hop-pickers are about the most grateful people one can nurse or do anything for. They appreciate all that is done for them. I had a sort of casualty department (in a parochial room) where I received patients twice a day, more frequently if occasion required. The pickers were glad enough to come for treatment or advice for their various ailments, often saying: "Ah, what a blessing it is that there are such places for us poor folk!" There were not many accidents. The cases were chiefly ulcerated legs, sore eyes, sprained wrists, and such-like, but poisoned feet and legs were rather common. I only heard of one death through the whole season. That was a case of diphtheria from a farm some distance away. The child, however, had been removed before my arrival. There were short periods when we had a fair amount of rain. This consequently brought cases of rheumatic colds, and the sufferers had to be confined to their huts and nursed on beds of straw. Occasionally I saw pillow-slips stuffed with straw, but looked in vain for sheets, and only now and then even heard of them. There was a woman with rheumatic fever who had to be removed to hospital, and a little girl with congestion of one lung, who afterwards showed symptoms of typhoid. In many instances people said that they had come down for the benefit of their health, and it so happened that often these were sufferers from chronic rheumatism or gout, who, instead of deriving benefit, as they had hoped, were either glad to return home or to remain all the time in their huts or tents. One woman with gout was, I believe, laid up all the three weeks, and supported by children who went out and worked during the day. On Sunday there was no "hopping." All was quiet until a late hour in the morning. It was the day for washing and a general cleaning up, and it is astonishing how tidy and clean some of them were in spite of the inconveniences under which they worked. Sunday was also the day for a general feeding up. Having obtained their week's money, or a portion of it the previous afternoon, they went out and did their Saturday night's shopping. This included chicken, sausages, chops, and various other things. Vegetables they often obtained from the country people round. I did sometimes meet people looking half-starved, but not often, most of the hoppers looked fairly well nourished. The weather varied a good deal, but on the whole it was not bad, and Sundays were generally fine. The days simply flew, and the end came all too quickly, so that I found myself wishing as I came home that it was always hop-picking time.

### Incipient Hip Disease.

### EXAMINATION QUESTIONS FOR NURSES.

Now that everybody is returning from rest and change, we must hope that our nurses are feeling refreshed and strengthened for their coming year of work and are enjoying that elasticity of body and mind without which work becomes a toil instead of a pleasure. In the monthly examinations which we are now beginning, I hope we shall see evidences of renewed energy and clear-headedness.

Let me remind you of a few things that are, unfortunately, often forgotten by candidates. First, more attention to rules is needed. Month after month papers are sent in with headlines, divisions, etc. . . . although such treatment of the subject is forbidden. Our space is limited, and for that reason all such headlines, divisions, and blocking in the papers cannot be permitted. Again, many dozens of answers come without name or address, and are of necessity at once ignobly entombed in the waste-paper basket.

These errors are careless oversights, but there are others of more consequence. First, there is frequently a tendency amongst competitors to wander from the main subject under discussion and go off as it were on the loop lines of platitude. This is a sign that the writer is not thoroughly at home in her subject, which presents some difficulty with which she does not know how to cope, and, like some hunted bird, she seeks cover in flowery bye paths, where she hopes the sportsman, as represented by the examiner, will not follow and drag into the searching light her verbose and lengthy paragraphs.

The last and most serious fault to which I would draw your attention is that of intruding into the domain of the medical man. Never attempt in nursing to settle points of importance or advise treatment. That is the doctor's sphere. Yours is to carry out all his orders loyally and to report to him exactly what has occurred. Do not give him your opinions; relate facts and leave him to draw his own conclusions. I mention this because the tone of some of the candidates' papers denotes a tendency to usurp the medical man's place and to be too liberal with theories and opinions.

The Examiner.

Question for October.

What symptoms in a child would cause you to suspect incipient hip disease, necessitating medical advice at once?

Rules.

The competition is open to all. Answers must not exceed 500 words, and must be written on one side of the paper only, without divisions, head lines, or marginal notes. The pseudonym, as well as the proper name and address, must be written on the *same* paper, and *not on a separate sheet.* Papers may be sent in for 15 days only from the day of the publication of the question. All illustrations strictly prohibited. Failure to comply with these rules will disqualify the candidate for competition. Prizes will be awarded for the best two answers. Papers to be sent to "The Editor," with "Examination" written on the left-hand corner of the envelope.

In addition to two prizes honourable mention cards will be awarded to those who have sent in exceptionally good papers.

N.B.—The decision of the Examiner is final, and no correspondence on the subject can be entertained.

Any competitor having gained three prizes within the current year shall be disqualified from taking another until 12 months shall have expired since the first prize was gained.

### Everybody's Opinion.

[Correspondence on all subjects is invited, but we cannot in any way be responsible for the opinions expressed by our correspondents. No communication can be entertained if the name and address of the correspondent are not given as a guarantee of good faith, but not necessarily for publication. All correspondents should write on one side of the paper only.]

### THE TESTIMONIAL TO MISS PETER.

Miss Amy Hughes writes from Queen Victoria's Jubilee Institute for Nurses, 120-122 Victoria Street: The arrangements about Miss Peter's testimonial are not yet decided. The Fund does not close till October 7. The Committee has not yet met to make arrangements. When it does, official intimation will be sent to the papers. Many suggestions are made, but nothing can be settled until the Committee has met.

### LOYALTY TO THE DOCTOR.

"Maternity Nurse" writes: In reply to "L. O. S. and C. M. B.," I should like to say that their experience is not uncommon. I have had 22 years' experience of maternity work, and I have met several cases which were a disgrace to medical practice. I know some doctors who always wash and scrub their hands every time they examine a patient, and others who will not waste time with water. I once sent for a doctor early in the morning, and he was going to the patient when I arrested him with "You have not washed your hands, doctor." He rejoined: "My hands are clean. I have just got out of bed." If we nurses were to do this we should be called dirty. I always take every precaution, and also carry with me a bottle of a well-known disinfectant wherever I go.

"Kentish L.O.S." writes: I am very pleased to see that some of the certificated midwives have taken up the matter of doctors and antiseptics. I can strongly endorse their statement, both by what I have seen and heard from nursing members of the Association. I will quote the words given by one or two in my present district. One woman, seeing a midwife scrub and disinfect her hands, remarked that when "Dr. —— attended me he had been gardening, and had all the dirt in his nails, but he did not scrub as you do." An L.O.S. with whom I am well acquainted, in charge of an abnormal case, found it necessary to ask the doctor to retire, as he was under the influence of drink. I would like to know how others would manage should this occur.

### DISTRICT NURSES AND POOR-LAW WORK.

"N. E." writes: I was interested to read your remarks headed "District Nurses and Poor-Law Work." I do not know exactly what the district nurse's duty is inside the Machynlleth Workhouse, but I know that the poor who receive parish relief outside the workhouse are nursed by the district nurse—that is, if they like to accept her services. I should be pleased to know more about the arrangement made by the Glanford Brigg Guardians. I myself do not approve of the district nurse doing "poor-law work," or, as you term it, "the duties which belong to the workhouse staff." I believe that is why the district nurse at Machynlleth resigned.

### NURSEMAIDS AND UNIFORM.

"A Trained Nurse" writes: Seeing a girl who was not a certificated nurse wearing the uniform of one, even to the veil, doing the cake-walk on a public road at Frinton-on-Sea, about a fortnight ago, I was perfectly disgusted, and now feel ashamed of my uniform instead of proud of it, as I have done hitherto. I think it abominable that these little nurse-girls should have the chance of abusing our hardly-earned dress. They appear to imagine that because they look after children, and are called nurses, that they are privileged to wear our uniform. What would one of these nursemaids do if, say, there were an accident on the road, and, seeing her near in uniform, she were called upon to lend first aid? I wish that something of the kind would happen to teach them the difference between certificated nurses and nursemaids. Cannot something be done to alter this state of things?

### ONLY A FEVER NURSE.

"Contented" writes: I quite agree with "Isolation Hospitaler" on the subject of training and appointments. One really wonders why so-called "trained" and "untrained" nurses should worry about their respective positions when they are able to obtain situations so eminently suitable to each. Although I have not had three years' consecutive training, during the time I was in hospital I passed my exams. equally with those who had been through the full time. Yet in applying for a post of superintendent nurse under Poor-law, although I could produce the certificate of training and had been nursing for twelve years (my surgical training had been of recent date, within five years of my application for this post), and had really good testimonials from doctors and matrons, because I had not had the recognised "three years' training" the guardians kindly told me that it was not in their power to elect me, as the Local Government Board would not accept experience for training. I have heard doctors repeatedly say that nurses whom I knew to have the three years' certificate, were useless in many cases where sympathy and tact were needed. So that I think we of the "Noble Army of Nurses," whether trained or otherwise, may be content to know that we are able to use our intellects and hearts for the benefit of suffering humanity wherever we may be placed; for, after all, it is as nurses we are called upon to act, not as doctors. It is impossible in these enlightened days to obtain responsible positions without having more than one testimonial of merit, and I do not think that doctors would give such to any woman unless she knew her work and did it well; and I think we adopt a narrow, bigoted view when we think we know more than others. We can but learn what we have opportunities of learning, and where is the nurse who knows all about everything?

### PROPOSED ALTERATION OF A SOUND RULE.

"Provincial Midwives" writes: With reference to the question of nurses practising midwifery alone, I should like to say that there are many parishes where there is not sufficient general nursing to occupy a nurse's whole time unless in combination with midwifery, especially when a patient resides a long distance from any doctor. Many women prefer to have a midwife as more fitting for them than a doctor, and unless abnormality occurred they would never call one in. Moreover, after several years' practice as nurse and midwife, I have met with no ill results, principally, perhaps, because I have always been very careful to use all necessary precautions. Again, other patients will return to the doctor because they prefer the dirty state in which the Gamp leaves them to the cleanliness of the midwife, hence the state of affairs in many parishes round my district. I would take this opportunity of asking my fellow midwives to leave the cottage neat and in order when they have found it so, and even when they have not to set the example. I frequently hear nurses require so much attendance, and leave soiled things about the room, so that it takes a neighbour a long time to attend to her. If this continues we shall never gain the day over Gamps. I always feel sorry when I learn that my predecessor, although possessing the L.O.S., has left things untidily, as it causes unnecessary prejudice. We ought not to make more work by leaving the bedroom untidy, unclean. The neighbour in poor districts, as well as servants in large households, are making complaints against the nurse on this score.

### "AN ISOLATION HOSPITAL WITHOUT A TRAINED NURSE."

"R. A. L. W." writes: I was pleased to see "M. C. I's" letter in your issue of last week in reply to mine of the previous week. Samuel Johnson once wrote: "Knowledge is of two kinds: we know a subject ourselves, or we know where we can find information on it." From the tone of "M. C. I.'s" latest letter, it is very clear that when she first wrote she neither knew her subject, nor did she make any inquiries as to her outspoken statements. Since that time she has seen the error of her way, and her reply last week shows that "a little knowledge is a dangerous thing." "M. C. I." wrote to you in her first letter that "the assistant nurses did not know how to clean the tube," and when I took her to task on this especial utterance, she writes: "I do not wish to infer that only trained nurses know how to remove, clean, and replace a tracheotomy tube." Both these statements flatly contradict each other. No, no. She did not infer—she all too plainly said what she is now trying to retract. With reference to the concluding portion of her letter, I do wish to compare Metropolitan Asylums Board nurses to the nurses of the isolation hospitals. "M. C. I." speaks of why should they be different to the M.A.B. nurses? The very fact of its being an Isolation Hospital shows clearly that it is for diseases of an infectious nature, so why the staffs of each should not have the identical knowledge of tracheotomy and the other branches I fail to see. My chief reason for inquiring of "M. C. I." the salary she received and the distance she had to go was simply because I, with others who are interested in the subject, have, to put a fine point on the matter, reason to doubt her statements. Where can "M. C. I." find a Board offering £40 to a caretaker, as your correspondent calls the matron of her special Isolation Hospital? "M. C. I." should know, if she be a trained nurse, that it would be a poor outlook for the patient if she had to be sent for ere anything could be done for the sufferer. I am afraid that unless "M. C. I." gives us fuller details we shall have to take her varying statements *cum grano salis*. In answer to your other correspondent, "A Fever Nurse," I am entirely at one with her; but she must not think that I fancy that all that is required in nursing a tracheotomy case is to abstract, clean, and replace the tube. I had merely mentioned this part of the treatment because "M. C. I." had endeavoured to make a point of it. We fever nurses are under a debt of gratitude to the writer of the editorial note for his timely remark on "Only a Fever Nurse." Were it not for nurses who are fever-trained it would be indeed a black outlook for not only London, but the provinces.

### PROBATIONERS AND THEIR PLEASURES.

"Matron R. R. C." writes : There is one lesson that some young nurses learn in a few months and others take a year or more to master, but it is one that has to be grasped by every hospital nurse at some time or other, and that lesson is never to make any appointment for off-duty time and then really set their heart upon being able to keep it for certain. The sooner they learn when making any arrangement for their own pleasure (or their own business) to add to the usual proviso of "D.V. and weather permitting," the addition of "matron and hospital duty permitting," the happier they will be in the course of their training, and their contented resignation will be reflected on their patients and on their fellow-workers. It is many years ago now, but I still remember one or two of my disappointments about being unable to carry out my plans when I was a young probationer. I once went to Henley Regatta with three brothers, one a solemn Colonial judge, another a solicitor, and the third a young curate. It was a gorgeous summer day, and we had a grand time. Moreover, the way these men threw away all their dignity and ran and shouted when their College boats came along was most refreshing. The next year I was a probationer at Great Ormond Street, and two brothers again asked me to go to Henley, and I was to choose which day would suit me best. I had been nearly a month in my ward and my "day off" was about due. I went to the sister and asked which day it would be convenient for me to have, telling her why I wanted to know. She was quite willing, and the date was all settled. The sister told the matron which day she was arranging to give me. A little thought and money had to be expended on my toilet, as brothers are rather particular, and it seemed as though fine weather was all we needed. The very morning before the Regatta the matron moved me to another ward, where there were several bad cases, and as they were already short handed, a nurse being away for her holiday, it was quite impossible for me to get the day off from there. I said nothing, but I know my old sister went and pleaded that I should not be moved for another week or even for a day or two, but it was no use. Neither she nor I could see any reason in it, and she told me afterwards that the probationer who was sent to her had been reported by the sister to whose ward I was sent, and so the matron moved her. But it did seem hard that she should get my day off and I had to have hers a week later, when I did not care twopence for it. And my brothers' tickets for the journey, the enclosure, and the lunch were all wasted. Of course, in looking back, this does not seem so bad as it did at the time, but hard and unjust treatment rankles in one's mind for a long time; and though, perhaps, it broke me in more rapidly to the habit of not being able to reckon upon anything outside my work, I feel now as I felt then, that girls should not be treated without due consideration, providing that they work heartily and well when on duty. The incident has made me very careful ever since I have had the control of young nurses to be very just in my dealings with them.

### Appointments.

[No charge is made for announcements under this head, and we are always glad to receive and publish appointments. The information, to insure accuracy, should be sent from the nurses themselves, and we cannot undertake to correct official announcements which may happen to be inaccurate. It is essential that in all cases the school of training should be given.]

Birkenhead Union Infirmary.—Miss Sarah Heap has been appointed charge nurse. She was trained at Burnley New Infirmary, and has taken sister's duty during holiday. She is registered under the Central Midwives' Board.

Bolingbroke Hospital, Wandsworth Common, London.—Miss Maud Bullock has been appointed sister. She was trained at the Chesterfield and North Derbyshire Hospital, and has since been staff nurse at Luton Cottage Hospital, staff nurse at Bromsgrove, Droitwich and Redditch Joint Isolation Hospital, and sister at the North Devon Infirmary, Barnstaple.

Bristol Infirmary.—Miss E. F. Mann has been appointed sister-in-charge of medical wards. She was trained at St. Thomas's Hospital, London, and has since been sister and night superintendent at Monsall Fever Hospital, Manchester.

Bromley Sick Asylum.—Miss Florence Farrow has been appointed second assistant matron. She was trained at Huddersfield Infirmary, and has since been night superintendent at Shoreditch Infirmary, staff nurse at Ramsgate Seamen's Hospital, and Huddersfield Infirmary.

City Hospital East, Old Swan, Liverpool.—Miss Grace Elliott has been appointed charge nurse. She was trained at Nottingham General Hospital, and has since been staff nurse at the Nursing Home, Nottingham; staff nurse at St. Mary's Hospital, Paddington; and assistant nurse at the City Hospital East, Old Swan, Liverpool.

East Sussex Hospital, Hastings.—Miss Ruth Thompson has been appointed staff nurse. She was trained at the Jessop Hospital, Sheffield, and at the Sheffield Royal Infirmary, where she has since been staff nurse and sister.

Folkestone Sanatorium.—Miss Laure H. de Bussy has been appointed matron, Miss Blanche E. Flack charge nurse, and Miss Helen Withers staff nurse. Miss de Bussy was trained at the Folkestone Sanatorium, and has since been staff and charge nurse at the Sanitary Hospital, Boscombe, Bournemouth. Miss Flack was trained at the Sanitary Hospital, Boscombe, Bournemouth, and Miss Withers at the Children's Hospital, Birmingham.

Fulham Poor-law Infirmary.—Miss E. M. Barber has been appointed midwife. She was trained at Kingston Infirmary, Kingston-on-Thames, and has since been ward sister. She has also been nurse at the Park Fever Hospital, Lewisham. She holds the certificate of the Central Midwives Board.

Hampstead General Hospital.—Miss A. d'Arcy has been appointed home sister of the Nursing Institute. She was trained at Sussex County Hospital, Brighton, and has since been night sister at Wolverhampton General Hospital. She has also been at the Palmer Memorial Hospital, Jarrow-on-Tyne.

Leamington Home for Incurables.—Miss M. O. Cruning has been appointed sister. She was trained at Liverpool Hahnemann Hospital, and has since had charge of the theatre at the Children's Infirmary, Liverpool; been charge nurse at the City Hospital, Liverpool, and charge nurse at the City of London Infirmary, Bow.

Mount Vernon Hospital for Consumption (Northwood Branch).—Miss Hope Ranson and Miss Bertha McDougall have been appointed sisters. Miss Ranson was trained at St. George's Hospital, Bombay; and Miss McDougall at the London Temperance Hospital.

Portsmouth Union Infirmary.—Miss Mary Hartley has been appointed assistant matron. She was trained at the Bradford Union Hospital and has since held the position of ward sister and night superintendent at Portsmouth Union Infirmary.

Salisbury Workhouse Infirmary.—Miss Bessie Anscombe has been appointed superintendent nurse. She was trained at Steyning Union Infirmary, and has since been charge nurse at Pontypridd Union and night charge nurse at Eastville Union, Bristol.

### Notes and Queries.

**REGULATIONS.**

**The Editor is always willing to answer in this column, without any fee, all reasonable questions, as soon as possible.**

**But the following rules must be carefully observed.**

**1. Every communication must be accompanied by the name and address of the writer.**

**2. The question must always bear upon nursing, directly or indirectly.**

**If an answer is required by letter a fee of half-a-crown must be enclosed with the note containing the inquiry.**

*Diphtheria.*

(1) Can you inform me where I can get a publication on the treatment of diphtheria, and the price?—*A. G.*

"A Practical Treatise on Diphtheria," by Dr. B. R. Martin, 2s.; "On Diphtheria: its Nature, Varieties, Pathology, Diagnosis, and Treatment," by Dr. R. Hunter Semple, 2s. 6d., can be obtained from the Scientific Press, 29 Southampton Street, W.C. The nursing of diphtheria is, of course, found in any nursing text-book, such as Watson's "Handbook for Nurses."

*Registration.*

(2) I am at present training in a hospital containing 70 beds. The term of training is three years, and a certificate is granted at the end of that period. Can you tell me if the certificate from this hospital will enable me to become a registered trained nurse?—*H. B.*

At present there is no State Registration for nurses.

*Nursing Homes Abroad.*

(3) Will you kindly tell me the address of the Anglo-American Nursing Institution in Rome? Also the address of a good private nursing home in Cairo, and the best way to obtain work in Egypt?—*B. F.*

The address of the Anglo-American Nursing Home in Rome is 265 Via Nomentana. We never give names and addresses of private nursing homes. To obtain work in Egypt, advertise that you wish to accompany a patient wintering there, or the Matron of the Kasr-el-Ainy Hospital, Cairo, might give you some information if you wrote enclosing stamped envelope.

*Recognised Training School.*

(4) Can you tell me the terms on which probationers are received into ——— Hospital? Also whether that hospital is a recognised training school for nurses? I have looked in "The Nursing Profession," but it does not give any particulars. I shall be glad if you will answer my inquiries as soon as possible.—*C. E. S.*

You must write to the matron to know on what terms probationers are received in the hospital you name. A recognised training school should be a hospital containing, amongst other qualifications, not less than 100 beds. The institution you name has only 45 beds.

*Inspector of Midwives.*

(5) I am a certified Queen Charlotte's nurse, and hold the Central Midwives Board certificate, and wish to apply for a post as inspector of midwives, either in England or Wales. Will you kindly tell me where to apply? Can you also tell me of any nursing institute which engages maternity nurses who have not had general training?—*Babs.*

Watch our advertisement columns. The posts are advertised by local municipal authorities. As a rule nursing institutions prefer maternity nurses who have had at least some training in a general hospital. But you might advertise.

*Incurable.*

(6) I should be grateful if you could tell me where I could get votes to get a very sad case into a home for incurables. This woman is quite without money, and the hospitals have given up the case as incurable.—*Nurse T.*

Write first to the Secretary of the British Home and Hospital for Incurables, 72 Cheapside, London, E.C., or of the Royal Hospital for Incurables, Putney Heath, to know if the case is suitable. There is a list of homes for incurables in "Burdett's Hospitals and Charities."

**Handbooks for Nurses.**

| | Post Free. |
|---|---|
| "How to Become a Nurse: How and Where to Train." | 2s. 4d. |
| "Nursing: its Theory and Practice." (Lewis.) ... | 3s. 6d. |
| "Nurses' Pronouncing Dictionary of Medical Terms." ... | 2s. 0d. |
| "Complete Handbook of Midwifery." (Watson.) ... | 6s. 4d. |
| "Preparation for Operation in Private Houses." ... | 0s. 6d. |

Of all booksellers or of the Scientific Press, Limited, 28 & 29 Southampton Street, Strand, London, W.C.

### For Reading to the Sick.

A SONG OF COMFORT.

Not always have we sorrow, there are seasons
When buoyant joy dispels all dreams of ruth—
Times when our thoughts of sorrow seem but treasons
To king-like Truth.

Not always are we vext by cares and troubles—
Often the griefs of life appear no more—
Vanished, as on a lake the rain-drop bubbles,
When showers are o'er.

Not always feel we that our hopes are blighted;
A glad fruition will they often gain,
When we perceive the good are aye requited
Who conquer pain.

Not always should we grieve, each tribulation
Is sent to purify—to raise the soul,
To fit it for its glorious destination—
A heavenly goal.

*Mackenzie Bell.*

It is a matter of economy to be happy, to view life and all its conditions from the brightest angle. It expands the soul. It renews. It awakens. It is a far truer form of sympathy than that mistaken sense of communion with grief and suffering which holds our friends in misery instead of helping them out of it. It is a far nobler religion than that creed which causes one to look as serious as possible. There is something wrong in our philosophy if it sanctions melancholy and pessimistic thoughts. We have not yet looked deep enough into life. We have never got beyond being impressed by the sadder and gloomier side of life. We are still thinking and acting contrary to, not in harmony with, the happy world of nature by which we are surrounded. By maintaining this mournful attitude, we show our want of faith in the goodness of things as much as when we fear.

A deep, unquenchable spirit of joy is at once the truest evidence that we believe in the beneficence of the Father, and that we have penetrated deep enough into life's mystery to see how best, most courageously, and helpfully to take it. Every slightest experience will teach us something if we question it, and will yield its message of hope. This is the chief value of all experience; for the final meaning of suffering is hope, the last word of this final meaning of suffering is hope, as it speaks to us in moments of despair, in times of trouble, throughout life, throughout history, in all evolution.—*H. W. Dresser.*

When all Thy mercies, O my God,
My rising soul survey,
Transported with the view I'm lost
In wonder, love and praise! . . .
Ten thousand, thousand precious gifts
My daily thanks employ;
Nor is the least a cheerful heart
That tastes those gifts with joy . . .
Through all Eternity to Thee
A joyful song I'll raise!
For oh! Eternity's too short
To utter all Thy praise!

*Addison.*

